# miR-720 Regulates Insulin Secretion by Targeting Rab35

**DOI:** 10.1155/2021/6662612

**Published:** 2021-04-05

**Authors:** Chunting Lu, Dan Wang, Yunlu Feng, Lie Feng, Zejian Li

**Affiliations:** ^1^Science and Education Office, The First Affiliated Hospital, Jinan University, Guangzhou 510630, China; ^2^Department of Internal Medicine, South China Normal University Hospital, Guangzhou 510630, China; ^3^Department of Endocrinology, The First Affiliated Hospital, Jinan University, Guangzhou 510630, China; ^4^Medical Center of Stomatology, The First Affiliated Hospital, Jinan University, Guangzhou 510630, China; ^5^School of Stomatology, Jinan University, Guangzhou 510630, China

## Abstract

miRNAs pose a good prospect in the diagnosis and treatment of type 2 diabetes (T2D). This study is aimed at investigating whether miR-720 targets Rab35 to regulate insulin secretion in MIN6 cells and its molecular mechanism and the clinical value of miR-720 as a specific biomarker of T2D. Fifty-five samples of new diagnosis T2D patients and normal control were collected. Levels of miR-720, fasting blood glucose, insulin, and other indicators of glucose and lipid metabolism were determined. We increased and decreased the miR-720 expression using miR-720 mimic and inhibitor to identify the effect of miR-720 on insulin secretion in MIN6 cells, respectively. Then, we used miR-720 mimic, miR-720 inhibitor, and dual luciferase reporter gene assays to prove miR-720 which regulates insulin secretion by targeting Rab35 in MIN6 cells. In addition, we overexpressed and silenced the Rab35 gene to detect the expression of PI3K, Akt, and mTOR in MIN6 cells by RT-PCR and western blot. In this study, circulating miR-720 was significantly higher in the T2D group than the control group, and miR-270 was positive correlated with FBG, while negatively correlated with FINS. The overexpression of miR-720 inhibited insulin secretion, and miR-720 downregulation promoted insulin secretion. miR-720 regulated insulin secretion by targeting Rab35 in MIN6 cells. Compared with the control group, the expression of PI3K, Akt, and mTOR was significantly decreased by the overexpression of the Rab35 gene, while the silencing Rab35 gene could induce the expression of PI3K, Akt, and mTOR. Furthermore, miR-720 mimic could activate the PI3K pathway. We conclude that miR-720 may be a potential biomarker for the diagnosis of T2D. Increase of miR-720 reduced the Rab35 expression then activate the PI3K/Akt/mTOR signal pathway, thus inhibiting insulin secretion.

## 1. Introduction

Type 2 diabetes (T2D) is a homeostasis disorder of glucose metabolism caused by insulin resistance (IR) and/or pancreatic *β* cell dysfunction, which involves the interaction of genetic, environmental and behavioral factors [1, 2]. Previous studies on the pathogenesis of T2D mainly focused on pancreatic *β* cell function and insulin secretion regulation. However, recent studies have confirmed that epigenetic mechanisms play an important role in the pathophysiological process of T2D [3].

MicroRNA (miR) is a highly conserved non-coding small RNA widely distributed in eukaryotic cells, 18-25 nucleotides long, being capable of affecting the biological functions of other genes by binding to the 3′UTR end of its target gene [4, 5]. More and more evidences [6–8] show that miRNAs are involved in the regulation of glucose metabolism, insulin synthesis, and secretion, which are the core links in the development of T2D. There is no doubt that miRNAs provide new molecular clues for the pathological study of T2D, and miRNAs are expected to be specific diagnostic markers and therapeutic targets for T2D [9, 10]. miR-720 has been widely studied since its discovery, which is closely related to the pathophysiological process of various tumors. For example, the expression of miR-720 was decreased in breast cancer, and it was associated with lymph node metastasis [11]. The expression of miR-720 increased in cervical cancer [12]. In colorectal cancer, the expression level of miR-720 in tumor tissues is higher than that in normal tissues [13]. Compared with healthy people, the expression of miR-720 in serum of colorectal cancer patients was significantly increased, and its expression level was related to male gender and lymph node metastasis of colorectal cancer patients. Some studies suggest that miR-720 may be a prognostic marker for these specific types of cancer [14]. The research on miR-720 in T2D is still very limited yet.

Rab proteins are known to be important participants in insulin secretion by pancreatic *β* cells. Rab functions are accompanied by cyclical activation and inactivation of GTP-bound and GDP-bound forms between the cytosol and plasma membrane that are regulated by upstream regulators [15]. In our previous study [16], we found that Rab35 is closely related to the insulin secretion in pancreatic *β* cells, and Rab35 might be the target of miR-720 in pancreatic *β* cells.

Therefore, the aim of the current study was to investigate whether miR-720 targets Rab35 to regulate insulin secretion in MIN6 cells and its molecular mechanism and the clinical value of miR-720 as a specific biomarker of T2D. The results are expected to obtain reliable evidence that miR-720 regulates insulin secretion and provide a scientific basis for the application of miR-720 and its target as a new diagnostic index and treatment of T2D.

## 2. Materials and Methods

### 2.1. Ethical Compliance

This study was approved by the Ethics Committee of The First Affiliated Hospital of Jinan University, and written informed consents were provided by all the participants.

### 2.2. Experimental Materials and Reagents

We consecutively enrolled all consenting adult subjects (24–66 years old) with no past medical history who were seen for a health screening in the Endocrinology Department at the First Affiliated Hospital of Jinan University (Guangzhou, China) between January 2019 and July 2020. One hundred participants were recruited and classified into two groups: 50 healthy control and 50 T2D patients. The 50 healthy subjects in the control group were free from all endocrine diseases. T2D was confirmed in accordance with the World Health Organization (WHO) criteria [17]: fasting blood glucose (FG) levels ≥ 7.0 mmol/L or a 2 hour oral glucose tolerance test (OGTT) ≥ 11.1 mmol/L in the presence of symptoms and glycated hemoglobin (HbA1c) levels >6.5%. The 50 T2D patients were diagnosed for the first time, and no diabetes drugs were used before the diagnosis. The exclusion criteria were as follows: patients with other severe malnutrition; long-term use of glucocorticoids and other hormone drugs; patients with severe heart, liver, and kidney dysfunction; and patients with severe pneumonia, nephritis, and other systemic infectious diseases.

### 2.3. Reverse Transcription Polymerase Chain Reaction (RT-PCR)

About 10 ml venous blood was extracted, and total RNA was isolated using PAXgene Blood miRNA Kit (Qiagen) according to the manufacturer's recommendation. ABgene Reverse-iT One-Step RT-PCR Kit (ReadyMix Version), a Mastercycler gradient, and thermocycler (Eppendorf, Germany) using the following primers for miR-720 primers (forward primer, GCGTGCTCTCGCTGGGG; reverse primer, GTGCAGGGTCCGAGGT); PI3K primers (forward primer, AGCAGGTACCCTGGTGATTG; reverse primer, AGAAGGCACAGGTCCAGAGA); Akt primers (forward primer, ACTCATTCCAGACCCACGAC; reverse primer, CCGGTACACCACGTTCTTCT); mTOR primers (forward primer, GGGCAGCAACAGTGAAAGTG; reverse primer, ACGGAAGAAGCCTTGGACAG); and *β*-actin primers (forward primer, AAATCTGGCACCACACCTTC; reverse primer, GGGGTGTTGAAGGTCTCAAA) were synthesized [18].

### 2.4. Western Blotting

Western blot analysis was performed as described previously [5]. Briefly, proteins were separated using SDS-PAGE and electrophoretically transferred to polyvinylidene difluoride membranes. The membranes were blocked in Tris-buffered saline with 5% milk and 0.05% Tween and incubated overnight at 4°C with primary antibodies including rabbit anti-Rab35 (1 : 1000, USA), rabbit anti-PI3K (Abcam), rabbit anti-Akt (Abcam), rabbit anti-mTOR (Abcam), rabbit anti-GFP (Abcam), and rabbit anti-PTPRN2 antibodies (Sigma). After being washed 3-5 times with TBST, the membranes were incubated with horseradish peroxidase-conjugated goat anti-rabbit secondary antibodies (Jackson ImmunoResearch) and visualized using enhanced chemiluminescence reagents.

### 2.5. Cell Line and Culture

The mouse insulinoma cell line MIN6 was obtained from BoGu Biotechnology Co. Ltd. (Shanghai, China). High-glucose (4500 mg/L) Dulbecco's Modified Eagle's Medium (DMEM) was purchased from Thermo Fisher Scientific (Waltham, MA, USA). Fetal bovine serum (FBS) was purchased from Biological Industries (Cromwell, CT, USA). The MIN6 cells were maintained in high-glucose DMEM supplemented with 12% FBS, 10 *μ*l/L *β*-mercaptoethanol (Sigma-Aldrich, St. Louis, MO, USA), 100 U/ml penicillin, and 100 *μ*g streptomycin mixture (Solarbio, Beijing, China) at 37°C in 5% CO_2_.

### 2.6. Insulin Enzyme-Linked Immunosorbent Assay (ELISA)

After transfection for 48 h, supernatants from MIN6 cells were assayed for insulin. Insulin was quantified according to the manufacturer's protocol using 96-well plates and an Insulin Mouse ELISA Kit (catalog# EMINS, Invitrogen Corporation, CA).

### 2.7. Statistical Analysis

Results were shown as mean ± standard deviation (SD). The statistical analysis was performed using the SPSS 18.0 (SPSS Inc., Chicago, Illinois), and the data were visualized using the GraphPad 7. The independent *t*-test was employed for comparison between two groups. The correlation between miR-720 and FBG and miR-720 and FINS were assessed by one-way ANOVA, followed by Fisher's least significant difference test. Two-tailed *P* values <0.05 were considered statistically significant.

## 3. Results

### 3.1. Clinical Characteristics of the Subjects

Fifty healthy control subjects and 50 T2D subjects were enrolled, and the relevant clinical data were shown in Table 1. Gender and age distribution showed no statistical difference (*P* > 0.05) in two groups, whereas body mass index (BMI), fasting blood glucose (FBG), c peptide (C-P), fasting insulin (FINS), hemoglobin A1c (HbA1c), low-density lipoprotein cholesterol (LDL-C), high-density lipoprotein cholesterol (HDL-C), total cholesterol (TC), and triglyceride (TG) differed significantly in the T2D group when compared with the healthy control group.

### 3.2. Circulating miR-720 in Type 2 Diabetes

**Figure 1:**
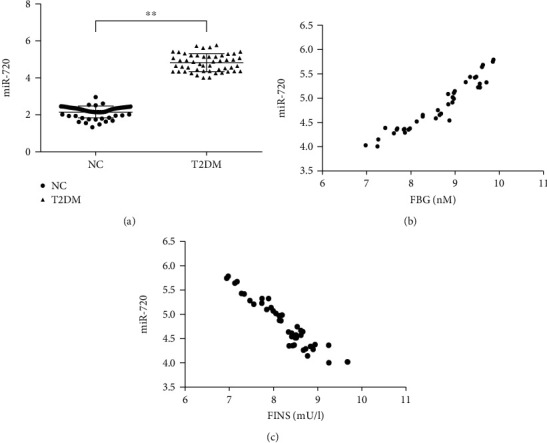
miR-720 was closely related to type 2 diabetes. (a) Circulating miR-720 in type 2 diabetes patients and normal participants, NC: normal control, T2D: type 2 diabetes, *n* = 50, respectively, ^∗∗^*P* < 0.01. (b) There is a positive correlation between miR-720 and FBG in T2D patients, *n* = 50, *r* = 0.95, *P* = 0.000. (c) The expression of miR-720 was negatively correlated with FINS, *n* = 50, *r* = −0.94, *P* = 0.000.

Circulating miR-720 was significantly higher in the T2D group compared with the control group (Figure 1(a)). There was a positive correlation between circulating miR-720 and FBG (Figure 1(b)). The expression of miR-720 was negatively correlated with FINS (Figure 1(c)). These results suggested that the secretion of miR-720 is closely related to T2D events.

### 3.3. miR-720 Effects on Insulin Secretion in MIN6 Cells

**Figure 2:**
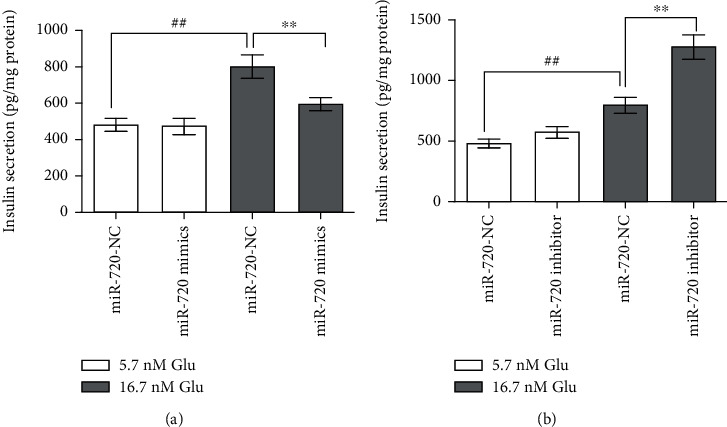
Effects of miR-720 on insulin secretion by MIN6 cells. Levels in MIN6 cells after transient transfection with miR-720 mimics (a) and inhibitor (b) under basal glucose (5.7 nM) and high-glucose (16.7 mM) stimulation. Data are shown as mean ± SD. ^##^*P* < 0.01, compared with the 5.7 mM glucose group. ^∗∗^*P* < 0.01, compared with the miR-720 NC group.

To identify the effect of miR-720 on insulin secretion in MIN6 cells, we increased and decreased the miR-720 expression using the miR-720 mimic and inhibitor, respectively, and detected the level of insulin secretion after stimulation with 5.7 and 16.7 mM glucose. The ELISA results showed that the miR-720 overexpression inhibited insulin secretion under high glucose stimulation (*P* < 0.01) (Figure 2(a)), and miR-720 downregulation promoted insulin secretion under high glucose stimulation (*P* < 0.01) (Figure 2(b)). Regardless of whether miR-720 was up- or downregulated, there was no effect on insulin secretion under basal glucose stimulation (Figure 2). These results indicated that miR-720 plays a negative regulatory role in GSIS, but not in insulin secretion at physiological blood glucose levels.

### 3.4. Rab35 Is the Target of miR-720

**Figure 3:**
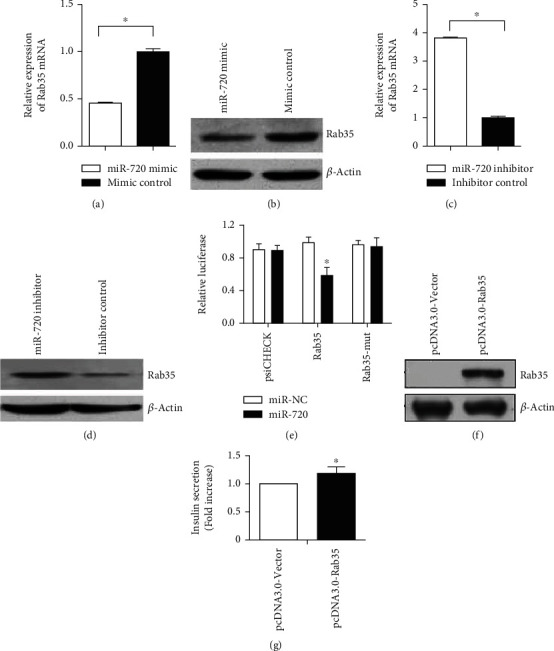
miR-720 regulates insulin secretion by targeting Rab35 in MIN6 cells. (a) miR-720 mimic could downregulate the Rab35 mRNA expression. (b) miR-720 mimic could downregulate the Rab35 protein expression. (c) miR-720 inhibitor could upregulate the Rab35 mRNA expression. (d) miR-720 inhibitor could upregulate the Rab35 protein expression. (e) Relationship between miR-20a and its target Rab35 is based on dual luciferase reporter gene assays. (f) Western blot showed that the constructed vector pcdna3.0-rab35 had a high expression level. (g) Compared with the control group transfected with empty pcdna3.0-vector, the Rab35 overexpression promoted insulin secretion. ^∗^*P* < 0.05.

miR-720 mimic could downregulate Rab35 mRNA and protein expression in MIN6 cells (Figures 3(a) and 3(b)); miR-720 inhibitor could upregulate Rab35 mRNA and protein expression in MIN6 cells (Figures 3(c) and 3(d)). Relationship between miR-720 and its target Rab35 is based on dual luciferase reporter gene assays. The results showed that the overexpression of miR-720 significantly inhibited the firefly luciferase activity of Rab35 wild type compared with the miR-NC group (*P* < 0.05). However, there was no significant effect on the firefly luciferase activity of Rab35 mutant (Figure 3(e)).

Subsequently, we wanted to investigate whether Rab35 had a negative effect on insulin secretion compared with miR-720 in MIN6 cells. Therefore, we constructed an miR-720 insensitive Rab35 expression vector (pcDNA3.0-Rab35). The vector only contains Rab35 coding sequence on the basis of pcdna3.0-vector to avoid the target of miR-720. Successful overexpression of Rab35 was evaluated by western blot (Figure 3(f)). Then, we analyzed the effect of the Rab35 expression on insulin secretion in MIN6 cells. Compared with the control group transfected with empty vector pcdna3.0-vector, the Rab35 overexpression promoted insulin secretion (Figure 3(g)). The results suggest that Rab35 has an opposite effect on insulin secretion in MIN6 cells compared with miR-720, which further suggests that Rab35 may be a functional target of miR-720 in MIN6 cells. It was confirmed that miR-720 regulate insulin secretion by targeting Rab35 in MIN6 cells.

### 3.5. Rab35 Regulates the PI3K/AKT/mTOR Signaling Pathway

**Figure 4:**
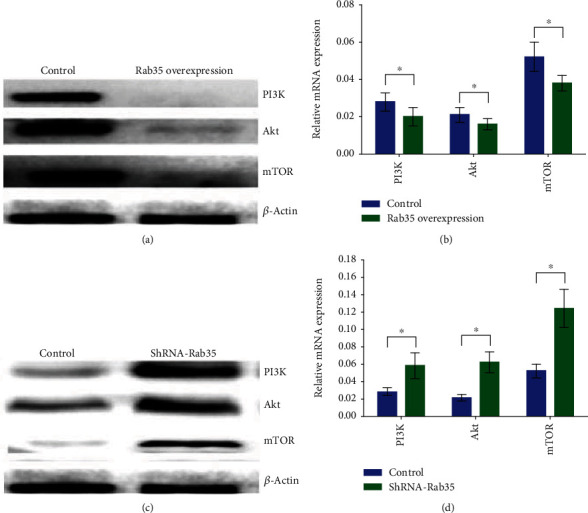
Rab35 regulates the PI3K/AKT/mTOR signaling pathway. (a). Western blot was used to detect the expression levels of PI3K, Akt, and mTOR after the Rab35 overexpression. (b). RT-PCR was used to detect the expression levels of PI3K, Akt, and mTOR after the Rab35 overexpression. (c). Western blot was used to detect the expression levels of PI3K, Akt, and mTOR after Rab35 silencing. (d). RT-PCR was used to detect the expression levels of PI3K, Akt, and mTOR after Rab35 silencing. ^∗^*P* < 0.05.

After overexpressed and silenced of the Rab35 gene, the expression of the PI3K/Akt/mTOR signaling pathway-related molecules in MIN6 cells was detected by RT-PCR and western blot, respectively. The results showed that compared with the control group, the expression of PI3K, Akt, and mTOR was significantly decreased by the overexpression of the Rab35 gene (Figures 4(a) and 4(b)), while the silencing Rab35 gene could induce the expression of PI3K, Akt, and mTOR (Figures 4(c) and 4(d)).

### 3.6. Increase of miR-720 Activates the PI3K/AKT/mTOR Signaling Pathway

**Figure 5:**
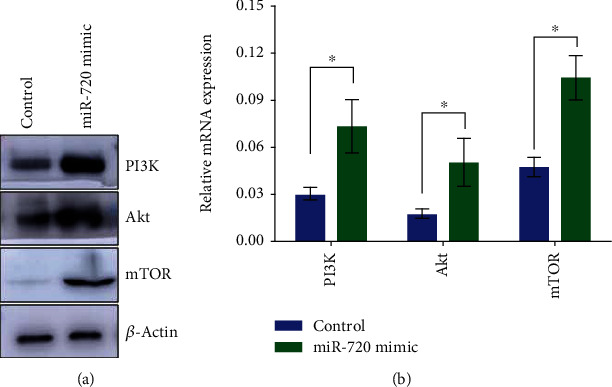
Increase of miR-720 activates the PI3K/AKT/mTOR signaling pathway. (a). Western blot was used to detect the expression levels of PI3K, Akt, and mTOR after miR-720 mimic transfection. (b). RT-PCR was used to detect the expression levels of PI3K, Akt, and mTOR after miR-720 mimic transfection. ^∗^*P* < 0.05.

In order to understand whether miR-720 increase may activate or inhibit the PI3K pathway, In MIN6 cells, the expression levels of PI3K/Akt/mTOR, mRNA, and protein were detected after miR-720 mimic transfection. The results showed that miR-720 mimic could upregulate the mRNA and protein expression levels of PI3K, Akt, and mTOR (Figure 5). This result suggests that miR-720 increase may activate the PI3K pathway.

## 4. Discussion

Circulating miRNAs have become ideal and noninvasive molecular markers to evaluate the pathophysiological status of various diseases due to their stable changes in diseases and stable existence in serum, plasma, urine, saliva, and other body fluids and easy to obtain and preserve clinically [19]. According to some previous studies, the levels of miR-126, miR-15a, and miR-223 in peripheral blood were decreased several years before the onset of T2D; compared with patients with DM and metabolic syndrome (MS), the serum levels of miR-9, miR-29, miR-30d, miR-34a, miR-124a, miR-146a, and miR-375 were higher in newly diagnosed T2D patients [20]; compared with the control group, the serum levels of miR-375 and miR-9 were higher in patients with prediabetes mellitus. These two miRNAs were directly related to the presence of prediabetes and T2D, and miR-375 was independently related to the occurrence of T2D. miR-375 alone or combined with miR-9 can be used as early detection markers of prediabetes and T2D [21]. In addition, studies have shown that miR-378, miR-126-3p, and miR-223 in blood are indicators of disease staging and prognosis in elderly patients with T2D [22]. In conclusion, serum miRNAs are noninvasive and ideal indicators for the diagnosis and prognosis of T2D [23]. However, no miRNA has been recognized as a specific diagnostic marker for T2D. In this study, we found that the serum miR-720 level of 50 newly diagnosed T2D patients was significantly higher than that of the healthy control group (*P* < 0.05), and miR-720 was positively correlated with the blood glucose level and negatively correlated with the insulin level (*P* < 0.05). This suggests that miR-720 is highly expressed in T2D patients and is related to blood glucose and insulin levels. This suggests that miR-720 may reflect the disorder of glucose metabolism in T2D patients to a certain extent and may become a potential biomarker for the diagnosis of T2D.

Insulin release from pancreatic *β* cells is a necessary condition for maintaining normal glucose homeostasis in humans and many other animals. miRNAs are known to be involved in the regulation of the insulin signaling pathway, but the mechanism is still unclear. Therefore, it may have important clinical value to explore the mechanism of miRNAs regulating insulin secretion by pancreatic beta cells and to understand the significance of miRNAs in T2D. Evidence suggests that miRNAs, as key regulators of the gene expression, play an important role in the production, transport, and secretion of insulin. The change of the miRNAs expression can lead to insulin secretion dysfunction and IR, destroy insulin signaling pathway and various physiological processes, and then lead to the occurrence and development of DM [24]. As a result, the potential role of miRNAs in the treatment of T2D has been widely concerned [25, 26].

The production of insulin is the key function of pancreatic beta cells, and the release of insulin is necessary to maintain glucose homeostasis. Among the known miRNAs that play an important role in insulin secretion and glucose homeostasis, miR-375 is the most concerned [27]. Some studies suggest that miR-375 is an important regulator of pancreatic beta cell function [28, 29]; the overexpression of miR-375 can reduce the number and viability of pancreatic beta cells [29, 30]. It can regulate the insulin secretion by directly targeting genes related to exocytosis [28, 31]. In addition, miR-103 and miR-107 can regulate insulin and glucose homeostasis in vivo. They play an important role in insulin sensitivity and may be potential targets for the treatment of T2D [20]. However, the mechanism of miR-720 in regulating insulin secretion by pancreatic beta cells is still unclear. Many studies have explored the function and target of miR-720 in disease progression. Known targets of miR-720 include TWIST1 in breast cancer [11], Rab35 in cervical cancer [12], StarD13 in colorectal cancer [13], and CCND1 in pancreatic cancer [14].

Other studies have shown that miR-720 can negatively regulate p63 and promote epithelial development [32]; miR-720 participates in the control of human dental pulp cell stem cell phenotype by directly inhibiting NANOG's level [33]; targeting miR-720 can help restore the immunity of patients with chronic hepatitis B [34]. Our study found that the overexpression of miR-720 inhibited glucose stimulated insulin secretion in MIN6 cells, while downregulation of miR-720 promoted insulin secretion. Subsequently, we found that Rab35 was the target of miR-720 in MIN6 cells by the luciferase reporter gene.

Rab proteins are also known to be important participants in exocytosis and secretion of insulin by pancreatic *β* cells. Rab functions are accompanied by cyclical activation and inactivation of GTP-bound and GDP-bound forms between the cytosol and plasma membrane that are regulated by upstream regulators [16, 35]. In our previous study, we found that Rab35 is closely related to the exocytotic and secretory function of pancreatic *β* cells [16]. The PI3K/Akt/mTOR signaling pathway regulates the life activities of many kinds of cells, including cell growth, proliferation, and differentiation. Overactivation of PI3K/Akt/mTOR is involved in diabetic retinopathy, diabetic nephropathy, and IR [36]. PI3K/Akt/mTOR is a signal pathway closely related to insulin signal transduction [6, 24]. The expression of PI3K and phosphorylation of Akt in the kidney, liver, skeletal muscle, and adipose tissue of DM rats was significantly decreased; in the development of DM, persistent hyperglycemia can promote the activation of the PI3K/Akt signaling pathway and ultimately accelerate the development of DM [37]. This study found that the overexpression of Rab35 inhibited the PI3K/Akt/mTOR signaling pathway and promoted insulin secretion; when Rab35 was inhibited, the PI3K/Akt/mTOR pathway was activated, and insulin secretion was downregulated. In addition, our further experimental results show that miR-720 mimic may activate the PI3K/Akt/mTOR signal pathway. Therefore, we speculate that miR-720 can target Rab35 and regulate the PI3K/Akt/mTOR signaling pathway thus affect insulin secretion.

## 5. Conclusions

**Figure 6:**
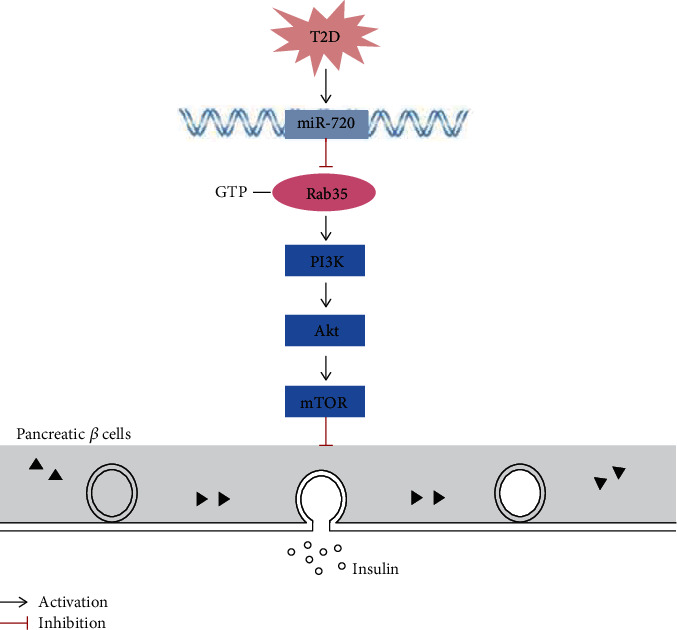
miR-720 regulates insulin secretion by targeting the Rab35/PI3K/AKT/mTOR signaling pathway.

Insulin is the most important hormone regulating glucose metabolism in vivo, and the normal secretion of insulin can maintain the homeostasis of glucose metabolism [38]. Studies at home and abroad have confirmed that miRNAs can regulate insulin secretion, and peripheral blood miRNAs can be used as biomarkers in the diagnosis and prognosis of T2D [21]. However, the research on miR-720 in T2D is still very limited. Therefore, this research fills the gap in this field to a certain extent and has research significance. In conclusion, we conclude that miR-720 may become a potential biomarker for the diagnosis of T2D; the increase of the miR-720 level can inhibit the Rab35 protein expression and then activate the PI3K/Akt/mTOR signal pathway related to insulin signal, thus inhibiting insulin secretion of pancreatic *β* cells (Figure 6).

## Figures and Tables

**Table 1:** General information of T2D patients and control group.

Factors	T2D group	Control group
Gender (M/F)	50 (24/26)	50 (27/23)
Age (years)	57 ± 8.2	55 ± 7.8
BMI (kg/m^2^)	26.2 ± 4.1^∗∗^	23.1 ± 3.8
FBG (mmol/l)	13.68 ± 2.81^∗∗^	4.96 ± 0.86
C-P (ng/ml)	2.52 ± 0.77^∗^	2.09 ± 0.72
FINS (mU/l)	13.15 ± 5.47^∗^	10.12 ± 6.41
HbA1c (%)	9.89 ± 2.74∗∗	3.21 ± 1.27
LDL-C (mmol/l)	3.28 ± 0.37∗∗	2.38 ± 0.68
HDL-C (mmol/l)	1.18 ± 0.61∗	1.37 ± 0.34
TC (mmol/l)	4.70 ± 0.52^∗∗^	4.12 ± 0.85
TG (mmol/l)	2.49 ± 1.06^∗∗^	1.50 ± 0.58

Note: data are presented as number (percentage) for categorical data or mean ± standard deviation (SD) for parametrically distributed data. Abbreviations: BMI: body mass index; T2D: type 2 diabetes; FBG: fasting blood glucose; C-P: c peptide; FINS: fasting insulin; HbA1c: hemoglobin A1c; HDL-C: high-density lipoprotein cholesterol; LDL-C: low-density lipoprotein cholesterol; TC: total cholesterol; TG: triglyceride. ^∗^*P* < 0.05 compared to the control group. ^∗∗^*P* < 0.01 compared to the control group.

## Data Availability

The data used to support the findings of this study are available from the corresponding author upon request.
